# Case report: Transient lactate elevation by intravenous insulin infusion therapy for diabetic ketoacidosis in a patient with mitochondrial DNA 3243 A > G mutation: A glycolysis rebooting syndrome?

**DOI:** 10.3389/fcvm.2023.1144925

**Published:** 2023-04-17

**Authors:** Wataru Ohwada, Hidemichi Kouzu, Tatsuya Sato, Kahomi Sazawa, Azumi Matsui, Nobutaka Nagano, Masayuki Koyama, Noriko Ogasawara, Akifumi Takada, Toshiyuki Yano, Masato Furuhashi

**Affiliations:** ^1^ Department of Cardiovascular, Renal and Metabolic Medicine, Sapporo Medical University School of Medicine, Sapporo, Japan; ^2^ Department of Cellular Physiology and Signal Transduction, Sapporo Medical University School of Medicine, Sapporo, Japan; ^3^ Department of Public Health, Sapporo Medical University School of Medicine, Sapporo, Japan; ^4^ Department of Microbiology, Sapporo Medical University School of Medicine, Sapporo, Japan; ^5^ Department of Otolaryngology-Head and Neck Surgery, Sapporo Medical University School of Medicine, Sapporo, Japan; ^6^ Department of Cardiology/Diabetic Internal Medicine, Steel Memorial Muroran Hospital, Muroran, Japan

**Keywords:** mitochondrial disease, cardiomyopathy, lactate metabolism, glycolysis, diabetic ketoacidosis, case report

## Abstract

Mitochondrial disease, most cases of which are caused by mitochondrial DNA (mtDNA) mutation, is present with multiple phenotypes including diabetes mellitus, sensorineural hearing loss, cardiomyopathy, muscle weakness, renal dysfunction, and encephalopathy, depending on the degree of heteroplasmy. While mitochondria play an important role in intracellular glucose and lactate metabolism in insulin-sensitive tissues such as muscles, appropriate strategies for glycemic control have not yet been established in a patient with mitochondrial disease, which is often complicated by myopathy. Here, we describe the history of a 40-year-old man with mtDNA 3243A > G who had sensorineural hearing loss, cardiomyopathy, muscle wasting, and diabetes mellitus with stage 3 chronic kidney disease. He developed mild diabetic ketoacidosis (DKA) in the process of treatment for poor glycemic control with severe latent hypoglycemia. According to the standard therapy for DKA, he was treated with continuous intravenous insulin infusion therapy, which unexpectedly resulted in an abrupt and transient elevation in blood lactate levels without exacerbation of heart failure and kidney function. Since blood lactate levels are determined by the balance between lactate production and consumption, an abrupt and transient lactate elevation following intravenous insulin injection therapy may reflect not only enhanced glycolysis in insulin-sensitive tissues with mitochondrial dysfunction but also decreased lactate consumption in the sarcopenic skeletal muscle and failing heart. Intravenous insulin infusion therapy in patients with mitochondrial disease may unmask derangements of intracellular glucose metabolism in response to insulin signaling.

## Introduction

Mitochondrial disease is mostly caused by an A > G transition at position 3,243 of mitochondrial DNA (mtDNA 3243A > G) encoding tRNA^Leu (UUR)^ gene, leading to respiratory chain complex dysfunction (1). Diabetes mellitus with mitochondrial disease, which accounts for about 1% of all diabetes (2), is a major phenotype of mitochondrial disease, although other complications such as sensorineural hearing loss, cardiomyopathy, muscle weakness, renal dysfunction, and encephalopathy are also present, depending on their proportion of cells in these organs with mutant mitochondria, called heteroplasmy (3, 4). Mitochondria play a pivotal role in intracellular glucose metabolism and the pathophysiology of impaired glucose metabolism in mitochondrial disease is unique compared with other types of diabetes. Owing to its uniqueness in that mitochondria dysfunction is a primary driver for the pathogenesis, appropriate strategies for glycemic control in mitochondrial disease have not been established.

Lactate is the end product of anaerobic glycolysis, which is produced from pyruvate, reduced nicotinamide adenine dinucleotide (NADH), and proton (H^+^) *via* the action of lactate dehydrogenase. Lactate can be produced even in aerobic conditions in the presence of mitochondrial disorders due to the impairment of pyruvate oxidation. Indeed, the ratio of lactate to pyruvate in the blood is known to increase, sometimes complicated by lactic acidosis, in patients with mitochondrial disease. An alternative mechanism of aerobic lactate production is enhanced glycolysis, which is typically observed in cancer cells, known as the Warburg effect (5). Despite the close relationship between glucose metabolism and lactate production, evidence suggesting the link between anti-diabetic therapies and elevated blood lactate levels in patients with mitochondrial disease has been sparse, although biguanides, compounds acting as a mitochondrial complex inhibitor, are not generally recommended because of the potential risk of inducing lactic acidosis.

Here, we present a case history of a 40-year-old man with mitochondrial disease who presented with cardiomyopathy, muscle weakness, and diabetic ketoacidosis (DKA). He was treated with continuous intravenous insulin therapy according to the standard therapy for DKA, which was surprisingly accompanied by a rapid and transient increase in blood lactate level despite elevated blood ketone level was decreased. Considering that blood lactate levels are regulated by the balance between its production and consumption, a rapid and transient increase in blood lactate levels observed in this patient may reflect not only enhanced lactate production in insulin-sensitive tissues with mitochondrial dysfunction but also limited lactate consumption in the sarcopenic skeletal muscle and failing heart. This case report implicates the need for attention to changes in blood lactate levels during intravenous insulin infusion therapy in mitochondrial disease.

## Case description

A 40-year-old man who was diagnosed with mitochondrial disease caused by mtDNA 3243A > G at the age of 22 years was referred to our institution to undergo cochlear implantation. The patient was determined to be at high risk for general anesthesia with numerous unassessed mitochondrial disease-associated complications, including heart failure, and was admitted to undergo thorough medical examinations before the operation of cochlear implantation. His family history implicated that his mitochondrial disease was maternally inherited rather than *de novo* onset, as his mother had diabetes and died from heart disease at the age of 40, although it was difficult to trace detailed information because he has no children or siblings. His laboratory findings on admission are shown in Table 1. He had multiple complications of mitochondrial disease, including sensorineural hearing loss, cardiomyopathy with reduced left ventricular ejection fraction of 28% and increased serum N-terminal pro-brain natriuretic peptide (NT-proBNP) level of 2839 pg/ml on admission, and diabetes mellitus with stage 3 chronic kidney disease (estimated glomerular filtration rate: 30–59 ml/min/1.73 m^2^) with moderate albuminuria (urine albumin-to-creatinine ratio: 30–300 mg/gCr). Although his body mass index (20.2 kg/m^2^) was not obviously decreased, a dual-energy x-ray absorptiometry (DEXA) scan revealed a marked reduction of appendicular skeletal muscle mass index (ASMI: 5.34 kg/m^2^, cut-off value of ASMI defined as ≤7.00 for Asian men in the diagnosis of sarcopenia) and a handgrip strength test showed decreased grasping power (right: 22.6 kg, left: 19.4 kg, cut-off value of handgrip strength <26 kg for Asian men) (6), indicating that he was complicated with muscle wasting. As a treatment for diabetes mellitus, he was on basal/bolus insulin therapy (insulin aspart at a dose of 10 units before each meal and insulin degludec at a dose of 14 units/day). Although his hemoglobin A1c (HbA1c) on admission was 8.1% indicating poor glycemic control, continuous glucose monitoring (CGM) that was introduced after admission revealed multiple events of severe latent hypoglycemia with the onset of non-sustained ventricular tachycardia, which has been reported to be associated with severe hypoglycemia (7). Since his endogenous insulin secretory capacity was not completely depleted with fasting serum C-peptide level of 0.34 ng/ml, insulin aspart was tapered and insulin degludec was replaced with a fixed dose combination of insulin glargine and lixisenatide to minimize the possibility of hypoglycemia. He was then scheduled for a coronary angiogram. The night before the examination, 30 mg of prednisolone was administered in accordance with our hospital safety manual because he had a medical history of a bronchial asthma attack that had been treated and controlled with an inhaled steroid and a long-acting *β*2-adrenergic receptor agonist.

**Table 1:** Laboratory findings on admission.

Complete blood count			Diabetes-related measurements			Urine tests		
WBC	7700	/*μ*l	Fasting glucose	79	mg/dl	Urine glucose	++++	
Neutrocyte	69.0	%	HbA1c	8.1	%	Urine WBC	-	
Lymphocyte	21.0	%	Fasting C-peptide	0.34	mg/dl	Urine RBC	-	
Monocyte	8.1	%	Anti-GAD antibody	<5.0	U/ml	UACR	139.1	mg/gCr
Eosinocyte	1.4	%	Anti-insulin antibody	<0.4	%			
Basocyte	0.5	%						
RBC	439	×10^4^/μl						
Hb	14.1	g/dl						
Ht	41.5	%						
Plt	26.4	×10^4^/μl						
Biochemistry measurements						Arterial blood gas		
TP	6.8	g/dl	Ca	9.1	mg/dl	pH	7.42	
Alb	4.4	g/dl	CRP	<0.10	mg/dl	PaCO_2_	35.8	mmHg
T-bil	0.8	mg/dl	TC	169	mg/dl	HCO_3_^−^	22.7	mEq/L
AST	25	IU	TG	105	mg/dl	PaO_2_	94.5	mmHg
ALT	12	IU	HDL-C	71	mg/dl	Lactate	2.2	mmol/L
ALP*	93	IU	LDL-C	77	mg/dl			
LDH*	333	IU	TSH	2.25	μIU/ml			
BUN	24	mg/dl	FT3	2.96	pg/ml			
Cr	1.16	mg/dl	FT4	1.05	ng/dl			
eGFR	54.6	ml/min/1.73 m^2^	ACTH	18.9	pg/ml			
UA	8.1	mg/dl	Cortisol	11.8	μg/dl			
Na	143	mEq/L	NT-proBNP	2839	pg/ml			
K	4.4	mEq/L	Pyruvate	1.6	mg/dl			

WBC, white blood cell; RBC, red blood cell; Hb, hemoglobin; Ht, hematocrit; Plt, platelet; HbA1c, hemoglobin A1c; Anti-GAD antibody, anti-glutamic acid decarboxylase; UACR, urine albumin-to-creatinine ratio; TP, total protein; Alb, albumin; T-bil, total bilirubin; AST, aspartate aminotransferase; ALT, alanine aminotransferase; ALP, alkaline phosphatase; LDH, lactate dehydrogenase; BUN, blood urea nitrogen; Cr, creatinine; eGFR, estimated glomerular filtration rate; UA, uric acid; Na, sodium; K, potassium; Ca, calcium; CRP, C-reactive protein; TC, total cholesterol; TG, triglycerides; HDL-C, high-density lipoprotein cholesterol; LDL-C, low-density lipoprotein cholesterol-cholesterol; TSH, thyroid-stimulating hormone; FT3, free thyroxine 3; FT4, free thyroxine 4; ACTH, adrenocorticotropic hormone; NT-proBNP, N-terminal pro-brain natriuretic peptide.

^*^ Tested by International Federation of Clinical Chemistry and Laboratory Medicine method.

The following day, he developed hyperglycemia (blood glucose: 547 mg/dl) and mild acidemia (blood pH: 7.33) with nausea and thirst. His scheduled coronary angiogram was postponed. His vital signs were blood pressure of 95/78 mmHg, pulse rate of 92 beats/min, and oxygen saturation of 97% with room air. He did not present with heart failure symptoms such as cold extremities or dyspnea. The results of the blood test are shown in Table 2. The patient had elevated serum levels of potassium (6.7 mEq/L) and *β*-hydroxybutyrate (1.3 mmol/L), but there was no apparent deterioration of renal function, such as oliguria or the significant elevation of serum creatinine levels. Although his blood lactate level (2.9 mmol/L) was slightly higher than the reference value (∼1.8 mmol/L), a marked difference was not observed compared to the previous value of 2.2 mmol/L. Based on these findings, the patient was diagnosed with mild DKA, and continuous intravenous insulin was immediately started in accordance with the standard therapy for DKA (8). Sodium bicarbonate was also administered to restore acidemia and hyperkalemia (9).

**Table 2:** Laboratory findings at the onset of DKA.

Complete blood count			Diabetes-related measurements			Urine tests		
WBC	10 900	/μl	Total ketone body	1.74	mmol/L	Urine glucose	++++	
Neutrocyte	93.9	%	*β*-hydroxybutyrate	1.30	mmol/L	Urine WBC	-	
Lymphocyte	5.3	%	Acetoacetate	0.29	mmol/L	Urine RBC	-	
Monocyte	0.4	%	Glucose	547	mg/dl			
Eosinocyte	0.2	%						
Basocyte	0.2	%						
RBC	447	×10^4^/μl						
Hb	14.3	g/dl						
Ht	41.0	%						
Plt	22.9	×10^4^/μl						
Biochemistry measurements						Arterial blood gas		
TP	7.1	g/dl	Ca	9.9	mg/dl	pH	7.33	
Alb	4.4	g/dl	CRP	<0.10	mg/dl	PaCO_2_	34.8	mmHg
T-bil	1.0	mg/dl	TC	149	mg/dl	HCO_3_^−^	18.0	mEq/L
AST	20	IU	TG	106	mg/dl	PaO_2_	93.3	mmHg
ALT	14	IU	HDL-C	57	mg/dl	Lactate	2.9	mmol/L
ALP*	89	IU	LDL-C	71	mg/dl			
LDH*	247	IU						
BUN	46	mg/dl						
Cr	1.47	mg/dl						
eGFR	42.2	ml/min/1.73 m^2^						
UA	6.6	mg/dl						
Na	129	mEq/L						
K	6.7	mEq/L						

WBC, white blood cell; RBC, red blood cell; Hb, hemoglobin; Ht, hematocrit; Plt, platelet; TP, total protein; Alb, albumin; T-bil, total bilirubin; AST, aspartate aminotransferase; ALT, alanine aminotransferase; ALP, alkaline phosphatase; LDH, lactate dehydrogenase; BUN, blood urea nitrogen; Cr, creatinine; eGFR, estimated glomerular filtration rate; UA, uric acid; Na, sodium; K, potassium; Ca, calcium; CRP, C-reactive protein; TC, total cholesterol; TG, triglycerides; HDL-C, high-density lipoprotein cholesterol; LDL-C, low-density lipoprotein cholesterol-cholesterol.

*Tested by International Federation of Clinical Chemistry and Laboratory Medicine method.

**Figure 1:**
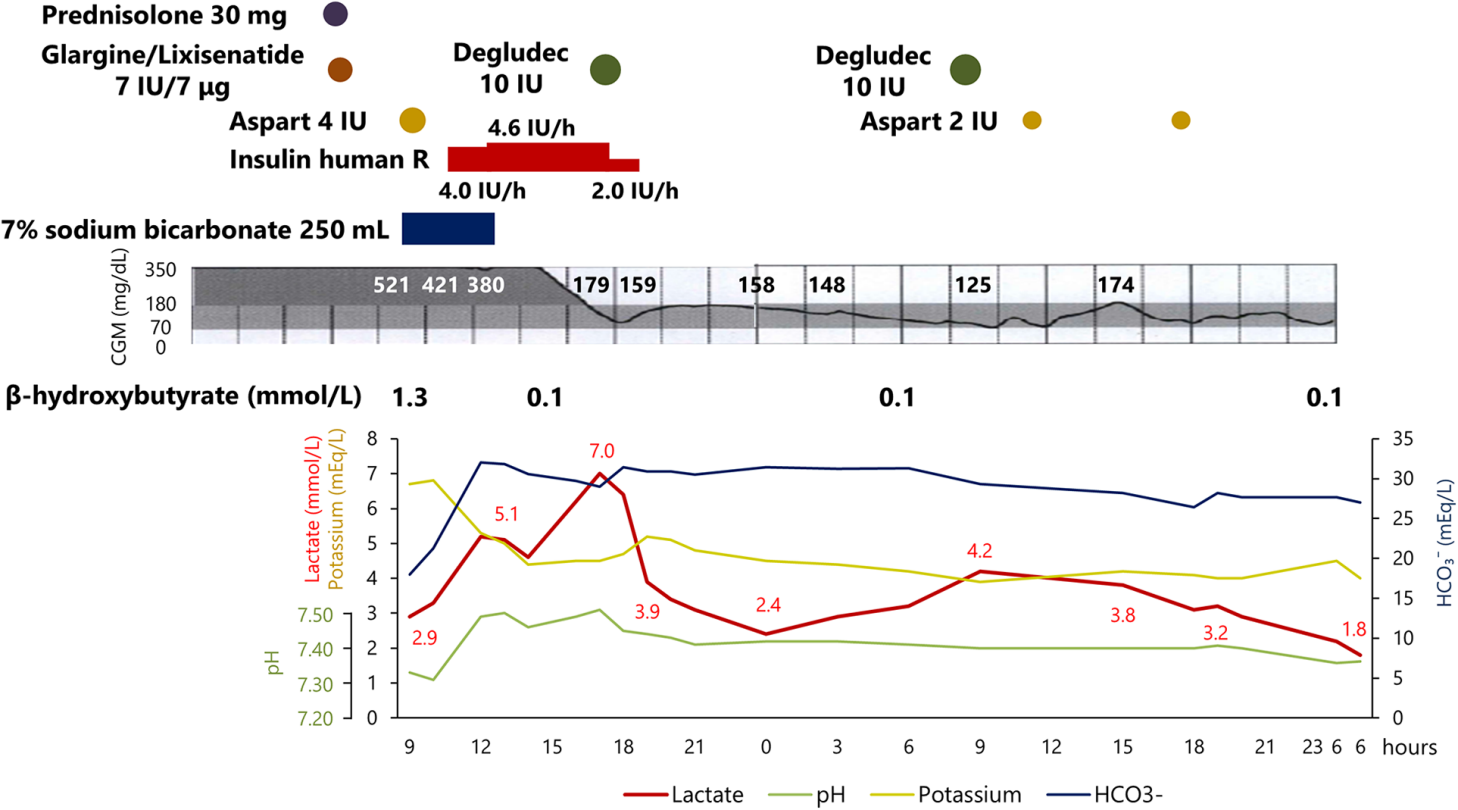
Time course of clinical features and treatment.

His clinical course after the onset of DKA is shown in Figure 1. Although the treatment quickly improved the hyperglycemia, ketosis, and acidemia, his blood lactate level was unexpectedly elevated to 7.0 mmol/L without exacerbation of his symptoms such as peripheral circulatory insufficiency caused by heart failure. Contrary to the elevated lactate level, there was no worsening of acidemia during the treatment. After switching from continuous intravenous insulin therapy to multiple subcutaneous insulin injection therapies with improvement in hyperglycemia, his lactate level began to decrease and was restored to the baseline level within 24 h. After that, his glycemic control was improved without recurrence of hyperlactatemia. He completed his planned cardiovascular scrutiny and was discharged without any sequela regarding the development of DKA and the transient lactate elevation.

## Discussion

Increased lactate production is induced by tissue hypoxia or uncoupling between glycolysis and mitochondrial oxidation, even under aerobic conditions. In mitochondrial disease, the latter mechanism causes blood lactate elevation at resting conditions, while the factors that induce lactate fluctuations remain unknown. Enhanced glycolysis by insulin in the insulin-sensitive tissues might provoke lactate overproduction in mitochondrial disease. However, although most patients with mitochondrial diabetes require insulin treatment (10), we could find only one previous report on the association between insulin therapy and hyperlactatemia, in which lactic acidosis developed during the treatment of DKA, as in the present case (11). Therefore, enhanced glycolysis alone seems insufficient to induce prominent hyperlactatemia in mitochondrial disease.

Lactate production and consumption must be balanced to maintain its blood concentration. The most well-known metabolic concept is that lactate produced by glycolysis in skeletal muscle is utilized for gluconeogenesis in the liver, known as the Cori cycle. The kidney also significantly contributes to gluconeogenesis from lactate, and lactate clearance is impaired in patients with acute kidney injury (12). However, our case showed neither liver failure nor acute kidney injury during the course. It has recently been demonstrated that most circulating lactate is oxidized as an energy substrate in all mitochondria-rich tissues (13). The rate-limiting reaction of lactate oxidation is its entry into the Krebs cycle *via* pyruvate dehydrogenase (PDH) after oxidation to pyruvate, and its activity is suppressed by NADH (14). Respiratory failure caused by mtDNA 3243A > G mutation leads to the accumulation of intracellular NADH, which correlates with the severity of mitochondrial disease (15). Therefore, the contribution of the affected organ to physiological lactate oxidation is expected to determine the circulating lactate level.

**Figure 2:**
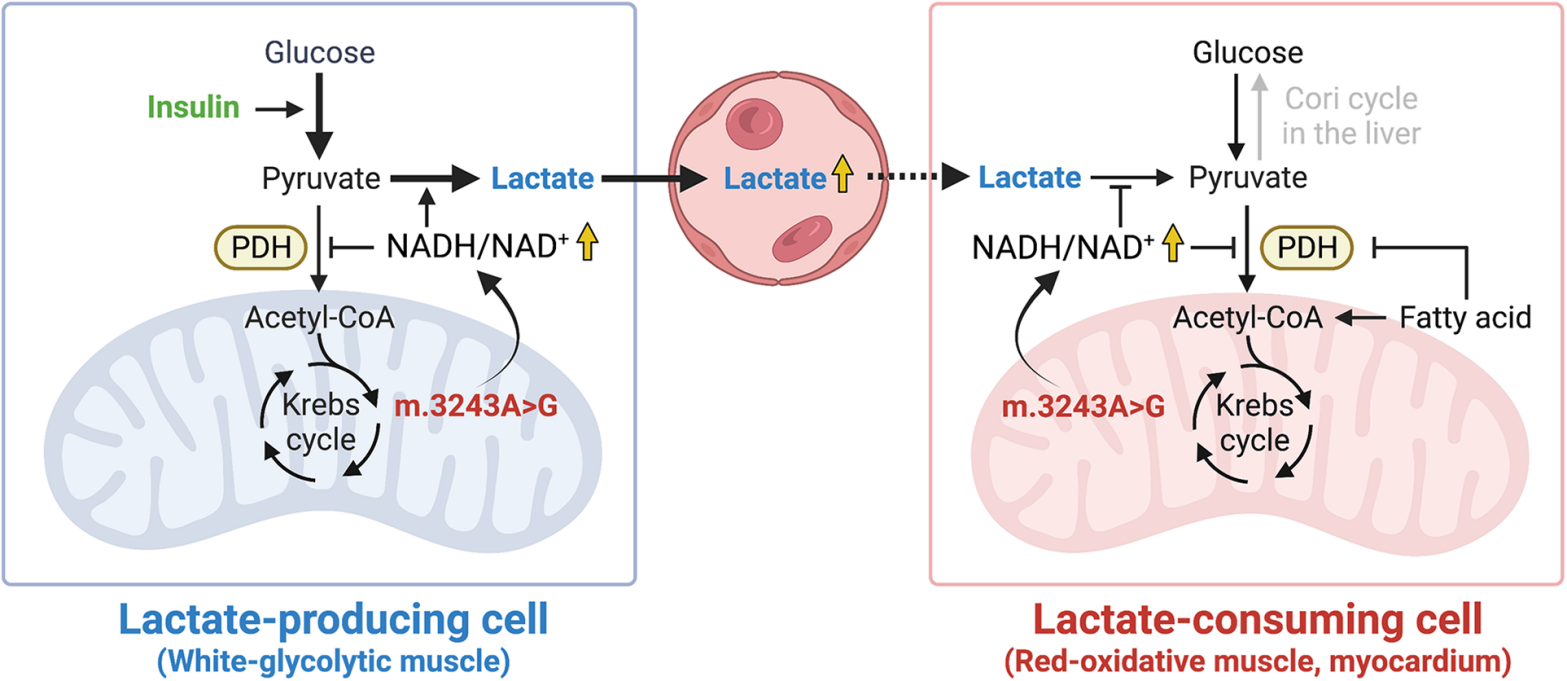
A schematic hypothesis. High NADH/NAD^+^ ratio induced by mtDNA 3243A > G mutation promotes the reduction of pyruvate to lactate in lactate-producing cells while it inhibits the oxidation of lactate to pyruvate in lactate-consuming cells. The illustration was created with BioRender.com. NADH, reduced nicotinamide adenine dinucleotide; NAD^+^, oxidized nicotinamide adenine dinucleotide; PDH, pyruvate dehydrogenase.

Muscle tissues are insulin-sensitive and are significantly involved in lactate metabolism. Within skeletal muscle, white-glycolytic fibers produce lactate, while red-oxidative fibers consume lactate (13). It was reported that skeletal muscle mass negatively correlated with the severity of mitochondrial disease (16). A DEXA scan and a handgrip strength test revealed that our patient was complicated with muscle wasting, likely contributing to hyperlactatemia accelerated by insulin infusion. In addition, since the myocardium is one of the major organs that oxidizes lactate (13), concomitant cardiomyopathy may also have contributed to impaired lactate clearance in our case. Taken together, these considerations not only suggest that lactate levels should be monitored during insulin therapy for mitochondrial disease, but also suggest that a marked lactate elevation with insulin administration can be a novel diagnostic test for mitochondrial disease in patients with decreased lactate consumption. Additionally, the DKA onset, likely triggered by insulin reduction and steroid administration, may have played an additive role in hyperlactatemia in our patient since activation of fatty acid metabolism during DKA suppresses PDH activity in muscle tissues (17). Furthermore, considerations should have been taken to prevent the development of DKA, such as avoiding the administration of prophylactic steroids, in this patient with mitochondrial disease. A schematic hypothesis for the mechanism involved in the transient blood lactate elevation in our patient is shown in Figure 2. Although elevated blood lactate levels are a conventional hallmark of mitochondrial disease, further research is needed to identify the factors that determine its variability.

In conclusion, careful monitoring for blood lactate levels should be performed when intensive insulin treatment is used for patients with mitochondrial disease. Future studies are needed to determine whether such a lactate spike is a clue to detect latent mitochondrial dysfunction by mtDNA mutation.

## Data Availability

The original contributions presented in the study are included in the article/Supplementary Material, further inquiries can be directed to the corresponding author/s.
